# Effectiveness of wetlands as reservoirs for integrated water resource management in the Ruzizi plain based on water evaluation and planning (WEAP) approach for a climate-resilient future in eastern D.R. Congo

**DOI:** 10.1038/s41598-024-72021-x

**Published:** 2024-09-16

**Authors:** Géant B. Chuma, Jean M. Mondo, Joost Wellens, Jackson M. Majaliwa, Anthony Egeru, Espoir M. Bagula, Prince Baraka Lucungu, Charles Kahindo, Gustave N. Mushagalusa, Katcho Karume, Serge Schmitz

**Affiliations:** 1 Faculty of Agriculture and Environmental Sciences, Université Evangélique en Afrique (UEA), Bukavu, South-Kivu Democratic Republic of Congo; 2 UR SPHERES, University of Liège, Liège, Belgium; 3 Doctoral School of Agroecology and Climate Sciences, Université Evangélique en Afrique (UEA), Bukavu, Democratic Republic of Congo; 4 Department of Agriculture, Université Officielle de Bukavu (UOB), Bukavu, Democratic Republic of Congo; 5 Faculty of Sciences, Université Officielle de Bukavu (UOB), Bukavu, Democratic Republic of Congo; 6 RUFORUM, Makerere University, Kampala, Uganda; 7 Department of Natural Resources Management, Faculty of Agricultural Sciences and Environment, University of Kinshasa, Kinshasa, Democratic Republic of Congo

**Keywords:** IWRM, WEAP, Ruzizi plain, Watershed, Water demand, Climate-smart agriculture (CSA), Environmental social sciences, Hydrology, Hydrology, Natural hazards, Climate sciences

## Abstract

It is widely predicted that climate change’s adverse effects will intensify in the future, and along with inadequate agricultural practices, settlement development, and other anthropic activities, could contribute to rapid wetland degradation and thus exert significant negative effects on local communities. This study sought to develop an approach based on the Integrated Water Resource Management (IWRM) in the Ruzizi Plain, eastern Democratic Republic of Congo (DRC), where adverse effects of the climate change are increasingly recurrent. Initially, we analyzed the trends of climate data for the last three decades (1990–2022). Subsequently, the Water Evaluation and Planning (WEAP) approach was employed on two contrasting watersheds to estimate current and future water demands in the region and how local wetlands could serve as reservoirs to meeting water demands. Results indicate that the Ruzizi Plain is facing escalating water challenges owing to climate change, rapid population growth, and evolving land-use patterns. These factors are expected to affect water quality and quantity, and thus, increase pressure on wetland ecosystems. The analysis of past data shows recurrence of dry years (SPI ≤  − 1.5), reduced daily low-intensity rainfall (Pmm < 10 mm), and a significant increase in extreme rainfall events (Pmm ≥ 25 mm). The WEAP outcomes revealed significant variations in future water availability, demand, and potential stressors across watersheds. Cropland and livestock are the main water consumers in rural wetlands, while households, cropland (at a lesser extent), and other urban uses exert significant water demands on wetlands located in urban environments. Of three test scenarios, the one presenting wetlands as water reservoirs seemed promising than those considered optimal (based on policies regulating water use) and rational (stationary inputs but with a decrease in daily allocation). These findings highlight the impact of climate change in the Ruzizi plain, emphasizing the urgency of implementing adaptive measures. This study advocates for the necessity of the IWRM approach to enhance water resilience, fostering sustainable development and wetland preservation under changing climate.

## Introduction

Water is a vital and precious natural resource essential to life, health, human dignity, and food production^1–3^. Water plays a fundamental role in most sectors, from political, social, cultural, to economic and environmental lives^4,5^. Water resources are not merely a renewable commodity; rather, they constitute a finite and irregular resource, an endless gift from the sky^6^. Besides, they are unevenly distributed in space and time and are subject to various pressures, both natural and anthropogenic^7^. The risks of depletion are possible and attributed to potential climatic hazards, pressure from population growth, inadequate policies for water use, evolving land use, and more importantly, urban, industrial, and agricultural developments. All these factors significantly contribute to increased withdrawals and degradation of water quality and ecosystems^7–9^.

To reduce the negative impacts of water resource management worldwide, the Global Water Partnership (GWP), a global network providing knowledge and capacity building for a sustainable water resource management (WRM), and the Dublin Conference (1992) promote the concept of Integrated Water Resource Management (IWRM)^10,11^. It is a systematic process for sustainable development, allocation, and monitoring of water resource use in the context of social, economic, and environmental goals^12^. Many regions in Africa, and specifically in the Sub-Saharan Africa (SSA), face significant water management challenges due to limited water resources, utilization policies, and environmental quality^13–16^. Indeed, during the twentieth century, water consumption increased twice as fast as the population^17–19^. In Central Africa basins, water supply is insufficient to meet the growing demand generated by demographic, economic, and climatic constraints^17–19^. Numerous efforts have been made in favor of necessary reforms towards IWRM; and the later has been adopted as the preferred water management approach^15^. However, limitations in its application and implementation exacerbate constraints and make IWRM difficult to achieve in a sustainable way.

In the Central African region, planning and managing water resources at the watershed scale is important despite limited documentation at appropriate temporal and spatial scales to formulate sound decision-making strategies^20^. In the Democratic Republic of the Congo (DRC), for example, despite the significant potential in water resources^21–23^, there are still constraints in their use and accessibility^24,25^. The main reason is the absence of a water management policy, rather based on the integrity of stakeholders. Furthermore, water reserves are managed neither sustainably nor under an ecosystemic and integrated approach, while the use of decision-support tools at large, medium, and small scales is necessary^25^.

The Ruzizi Plain, located in the eastern DRC, stands as a testament to the intricate interplay between natural ecosystems and the sustenance of human livelihoods^26^. This region, endowed with diverse wetlands, holds the key to a sustainable water future, particularly in the face of climate change impacts that manifest as erratic rainfall patterns, extreme weather events, and shifts in hydrological regimes^27^. Water scarcity exacerbated by climate change and anthropogenic pressures, casts its shadow over the Ruzizi Plain^26–28^. The hydrological dynamics of the region are intricately linked to seasonal rains, the undulating landscape, and the interconnected network of wetlands. However, these once-reliable water sources are under pressure due to climate change-induced alterations in precipitation patterns, increased evaporation, and high anthropogenic pressures. In fact, climate change is a key driver of water scarcity in the Ruzizi Plain; which experiences shifts in rainfall patterns, with some areas witnessing reduced precipitation and others facing more intense and sporadic rainfall events^26^. Rising temperatures contribute to increased evaporation rates, drying up surface water sources and stressing ecosystems that depend on consistent water availability^26^.

As the population grows rapidly in the Ruzizi Plain, urbanization brings with it increased water consumption for domestic, industrial, and agricultural purposes^8,28^. In the Ruzizi Plain, as in most part of the eastern DRC, unplanned urban expansion further compounds the challenge, placing strain on existing water resources and infrastructure.

Agriculture is a vital economic activity in the Ruzizi Plain^29^. In this region, agriculture is still based on traditional to semi-traditional farming practices, coupled with expansion of agricultural land that lead to increased water extraction for irrigation^30^. Inefficient water use and lack of sustainable agricultural practices exacerbate the strain on available water resources^26^.

Paradoxically, wetlands can act as natural reservoirs to alleviate the water scarcity issue^31,32^. Human activities, including drainage for agriculture and settlement, encroachment, and pollution, compromise the ability of wetlands to retain and release water, diminishing their role as regulators of the hydrological cycle^33^. In general, these ecosystems can be viewed as extensive water reservoirs that play a crucial role in mitigating unforeseen droughts while concurrently offering various ecosystem services^34–36^. Furthermore, communities relying on the water resources of the Ruzizi Plain endure the most of the water scarcity. Limited access to clean and reliable water jeopardizes health, sanitation, and agricultural productivity leading to high vulnerability, particularly in rural areas, that face increased challenges in securing water for daily needs. This region, marked by its intricate web of wetlands, rivers, and terrestrial ecosystems, faces an impending crisis as the demand for water intensifies and the impacts of climate change manifest. Implementing IWRM principles can enhance the efficient and equitable use of available water resources. It involves balancing competing water needs, considering ecological sustainability, and involving local communities in decision-making^37–39^.

Addressing water scarcity in the Ruzizi Plain necessitates a multifaceted approach, including, at the first point, mitigation and adaptation strategies that involve balancing competing water needs, considering ecological sustainability, and involving local communities in decision-making, and at the second point, develop and upgrade water infrastructure to endure climate variability. This includes efficient irrigation systems, rainwater harvesting, and the rehabilitation of wetlands to enhance water retention, and empowering local communities with knowledge about water conservation, efficient water use, and sustainable agricultural practices^40^. Overall, in the Ruzizi Plain, limitations exist regarding the future use of water and a planning strategy that take into account environmental conditions^26^.

Numerous tools have been implemented for water use assessment, of which the Water Evaluation and Planning (WEAP) approach is considered as one of the best decision-support tools in integrated and scalable planning, integrating the analysis of changes in water supply, demand, and management by stakeholders^11,41,42^. WEAP is also used to evaluate the effects of climatic hazards, hydrology coupled with land use, and infrastructure use to plan water usage and priorities for watershed-scale water management^11,42^. Such a tool could serve as a fundamental element to initiate a water resource management and planning policy in the Ruzizi Plain.

We propose to contribute to a sustainable integrated water management (IWRM) using the WEAP tool in the Ruzizi Plain, eastern DRC, considering two contrasting watersheds, namely the Luberizi and Mulongwe watersheds, as case studies to elucidate the effectiveness of wetlands as water reservoirs to cope with shortages resulting from climate change. Specifically, this study aimed to: (i) characterize watersheds in the Ruzizi Plain, (ii) assess the climatic constraints through the analysis of chronological data series, (iii) assess water demand (current, mid-century, and end of the century) from various sectors in the Ruzizi Plain, and (iv) evaluate the effectiveness of three water management scenarios. Several elements can be proposed to justify this study such as limited documentation on water management and evidence regarding the nature of climate disturbances in the Ruzizi Plain^26^, and the significant food production role played by the Ruzizi Plain, in terms of dairy, rice, and root and tuber crop productions^29,43^. Furthermore, this is the first study using the WEAP tool in water management in eastern DRC following typical scenarios.

## Methodology

### Study area

#### Location

**Fig. 1:**
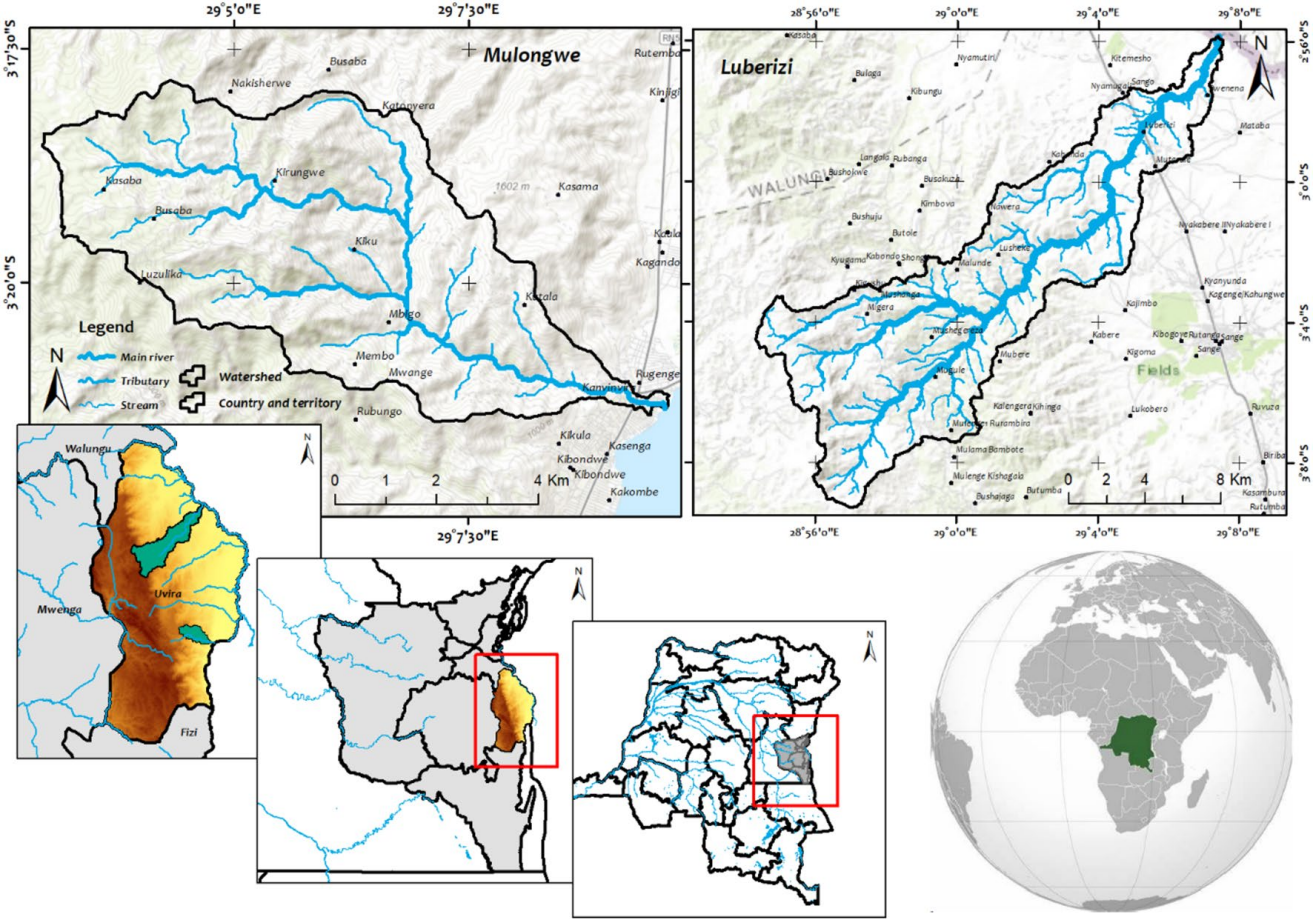
The Mulongwe and Luberizi watersheds in the Ruzizi plain, Uvira territory, eastern DRC.

The Ruzizi Plain is located in the Great Lakes region of the Central Africa, shared among Burundi, Rwanda, and the DRC^39^. The Ruzizi Plain part considered in this study is situated in eastern DRC, near the borders with Rwanda and Burundi. The plain is part of the broader Albertine Rift region and is characterized by its proximity to Lake Tanganyika^26^. The Ruzizi Plain in the DRC is characterized by its diverse landscape, influenced by its tropical climate, fertile soils, and a mix of grasslands and forests^43^. The region supports significant biodiversity and plays a crucial role in local ecosystems and agricultural production. In the next section, the two watersheds selected for this study, as case studies, are described separately (Fig. 1).

#### Climate

**Fig. 2:**
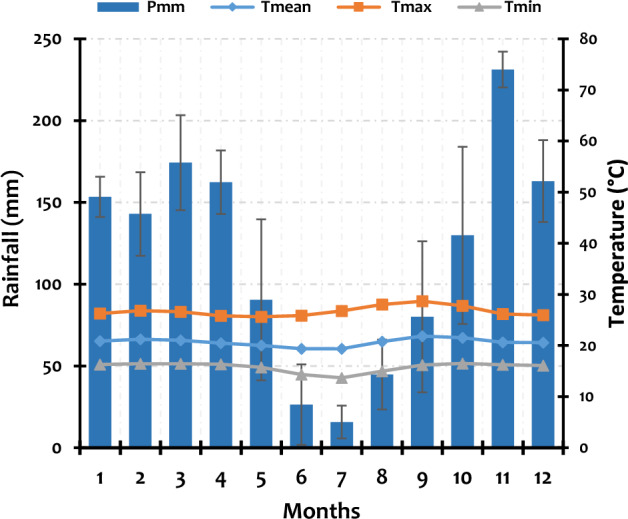
Climate data from local meteorological stations from 1990 to 2020 (the bar above each column represents the standard deviation of the mean monthly rainfall for the last 30 years).

The climate in the DRC’s Ruzizi Plain and in the two selected watersheds is typically semi-arid tropical, but influenced by its proximity to the Lake Tanganyika, the Mitumba mountain chain, and the Itombwe Natural Reserve. The region experiences a bimodal rainfall regime, with distinct wet and dry seasons. The wet season sees significant rainfall, while the dry season is marked by reduced precipitation^26^. Data from available weather stations revealed four consecutive dry months (June, July, August, and September), while May is occasionally dry and occasionally wet. November is the wettest month, followed by March, December, and April. The average temperature fluctuates at around 22 °C, with the maximum reaching ~ 30 °C and minimums of ~ 16–18 °C (Fig. 2). The climate contributes to the overall vegetation and agricultural production in the area.

#### Soil and topography

**Fig. 3:**
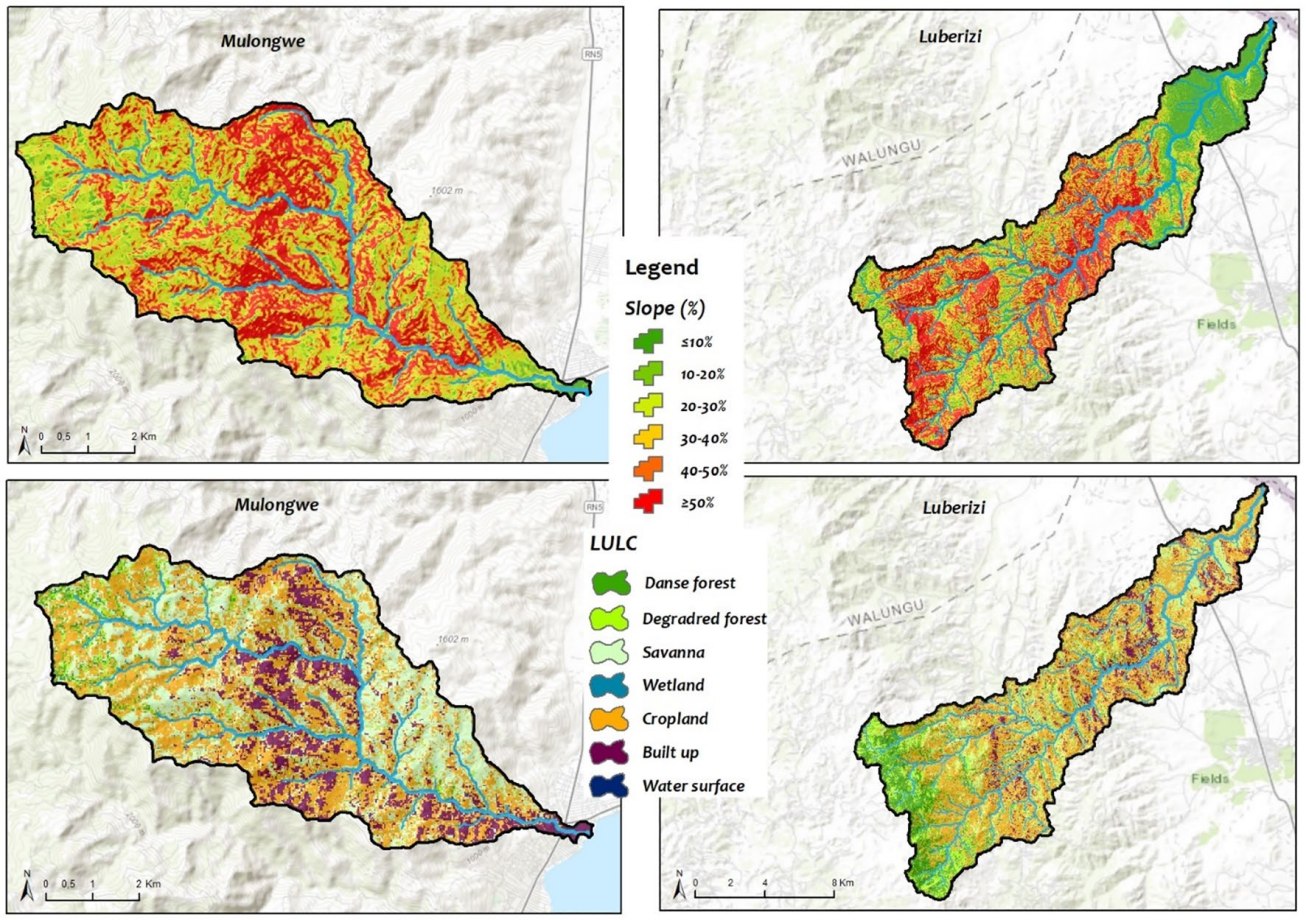
The Luberizi and Mulongwe watersheds in the Ruzizi plain, Eastern D.R. Congo (slopes were calculated using ALOSPALSAR DEM of 12.5 m resolution while the Land use and land cover (LULC) were determined using Landsat 8 OLI/TIRS data following the support vector machine (SVM) algorithm in ArcGIS 10.8.1 Esri-TM).

The two watersheds are characterized by steep slopes upstream, with the source of the main rivers located along the Mitumba mountain range, and downstream with very low slopes in the generally flooded valley flowing into the Ruzizi River or Lake Tanganyika. Such a structure affects the distribution of soil and hydrological phenomena in the watersheds. Soil textures for both watersheds range from sandy to sandy clay, characterized by varying clay levels and generally low organic matter (OM) and phosphorus contents. In the Luberizi watershed, Humic Ferrasols, Haplic Acrisols, and Luvic Phaeozem soils are prevalent, with Ferrasols constituting ~ 90% of the catchment^44^. The soils in the DRC part of the Ruzizi Plain are diverse, including fertile alluvial soils along the Ruzizi River, which are suitable for agriculture. Volcanic soils, stemming from the nearby Mitumba mountain range, also contribute to the region’s soil composition. These soils support a variety of crops and vegetation^39,45^. Ruzizi Plain area exhibits a rugged topography, ranging from 774 m above sea level (a.s.l.) at the Lake Tanganyika to 1460 m a.s.l in Mulongwe, and reaching an elevation of 3308 m a.s.l in Luberizi. This topographical variation is attributed to the East African Rift, an active Graben system that has been shaping the landscape for the past 30 million years. The drainage network closely follows these tectonic features (Fig. 3). The region is also characterized by active volcanoes along the rift structure, contributing to frequent landslides triggered by seismic activity. The geological composition predominantly consists of basaltic rocks dating back to the Cenozoic era. Additionally, Cenozoic sediments have accumulated in the Ruzizi River valley, particularly at its confluence with Lake Tanganyika^26,39,46^. This structure affects the distribution of soil and hydrological phenomena in the watersheds.

The vegetation includes a mix of grasslands, savannas, and patches of tropical forests (Fig. 3). The flora is adapted to the diverse soil types and climate conditions. Along the riverbanks and wetland areas, there are lush vegetation and wetland plants, such as reeds and papyrus^47,48^. The region’s flora supports local biodiversity and sustains various wildlife species. The Ruzizi Plain in the DRC is known for its rich biodiversity. It is a habitat for diverse bird species, mammals, and aquatic life^47^.

The analysis of satellite images on land use and land cover (LULC) of 2022 in the two selected watersheds, as well as in the entire Ruzizi Plain, reveals very low forest coverage, except for dense forests bordering the Itombwe National Reserve along the mountain peaks of the Mitumba mountain range. However, the area is dominated by degraded forests (mainly due to mining activities and tree logging for charcoal and timber), grassy savannas used as grazing areas for livestock, and agricultural fields primarily occupied by cassava and rice paddies. In some areas, wetlands are present, supplying water for rice cultivation (only inland valleys and floodplain and not marshes, swamps, peatlands, ponds, etc.), and grazing areas for animals during dry periods. In general, both watersheds are under significant anthropogenic pressures. In Luberizi, being an agricultural watershed, the development of agricultural activities (mainly rice cultivation, cassava, sweet potatoes, etc.) coupled with livestock rearing makes water demand a major concern. On the other hand, the Mulongwe watershed, located at the entrance of the Uvira city, faces intense anthropogenic pressure due to a high population growth rate, rapid urbanization, and pollution in various forms^21,39,49^. Further details on the methodology used for image classification are provided in our previous publication on land use and land cover that covered the entire South-Kivu province^50^. Control points were collected and trained samples were used for entrainment (70%) and 30% for validation. The classification was on Landsat 8 TIRS/OLI image downloaded from Earthexplorer.USGS.

### Methods

#### Characterization of Luberizi and Mulongwe watersheds in the Ruzizi plain, eastern DRC

To characterize these watersheds, a mixed method was used. This mixed approach helps to develop a holistic understanding of the watershed, which is essential for effective watershed management and conservation. First, geographic information system (GIS) tools combined with remote sensing technologies were employed in data collection and analysis. For each watershed, a description of various physical, hydrological, climatic, and land use aspects was done to assess its unique features. (i) The characterization started by defining watershed boundaries using the procedure proposed by Chuma et al.^51^. This was followed by analysis of the topography of the watershed in identifying high and low points, ridgelines, and drainage patterns. The information helped understand water flow within each watershed. (ii) Land use and land cover (LULC) followed, including areas under agriculture, settlement, forests, wetlands, etc. This information was crucial for assessing human impact on the watershed. This was combined with fieldwork for control point identification for image classification. The support vector machine (SVM) algorithm was used during satellite image classification in ArcGIS 10.8.1 Esri-TM. Field data samples were taken and split into training (70%) and validation control points following the methodology proposed by many studies^42,51,52^. (iii) The collection of hydrological and climate data such as stream flow, precipitation, evaporation, and other hydrological parameters helped to determine the flow paths and drainage patterns within the watershed. (iv) The assessment of the characteristics of soil and human activities in the watershed. The last step was identifying existing infrastructures and ecosystems (such as wetlands). As supported by the WEAP tutorial and relevant literature, water demand and supply and land use parameters were the most sensitive during parameterization.

#### Water hazard assessment based on climate data analysis in the Ruzizi plain

The assessment of the water hazard based on climate data analysis involves analyzing climatic conditions to determine potential risks and challenges related to water resources. This assessment typically included the analysis of climate parameters: precipitation patterns, temperature (maximum, minimum, means), wind speed, evapotranspiration, etc. Historical climate data from 1990 to 2022 were analyzed and helped identifying patterns and the potential impacts on water availability, quality, and overall water management in the Ruzizi Plain. The goal was to evaluate how climate variability and change may affect the water resources in the region, considering climatic factors and the frequency of extreme events. Such analysis was crucial for developing strategies to mitigate risks, adapt to changing conditions, and to ensure sustainable water management practices in response to climate change-related challenges are considered.

The RClimDex 1.9 package^53^ was utilized to assess climatic hazards in the study area. This package allows for the calculation of trends, correlations between climatic indices and time. The Sen’s slope method was employed to evaluate the correlation and trend of the chronological series of data^53^. Out of all the climatic indices provided by the package, only ten indices were considered (Supplementary Table 1). Additionally, the Standardized Precipitation Index (SPI) was calculated to identify months or years that are hotter or wetter, following the classes proposed by Tirivarombo et al.^54^. The description of these indices is presented in the Supplementary Table 1. To assess the annual trends of precipitation and temperatures, the Mann–Kendall test with a confidence level of 95% was used. With the Mann–Kendall test, two hypotheses were tested: the null hypothesis (H0): there is no trend in the time series, and the alternative hypothesis (H1): there is a significant trend in the time series, for α = 5%. The trend was quantified using the Sen’s slope method. Sen’s slope is an index used to quantify the trend using the non-parametric procedure^55^. This approach has been widely used in climate studies to provide quantitative inference on the strength of observed patterns^56,57^.

#### Water demand assessment for different actors in the two watersheds

The modeling of water demand relied firstly on the creation of different future scenarios based on water consumption^58^. The objective was to determine sectors where water demand is not met and estimate the quantity of water allocated to each sector. The LULC were merged and only four classes were retained comprising households, livestock, agriculture, and other urban users (such as markets, churches, and small industries, etc.). The choice of these different sectors was based on real-world knowledge of the environment during fieldwork. The approach proposed for water demand was based on a rather integrated method to assess and plan water resources. Water Evaluation And Planning (WEAP) has been used to assess the allocation of limited water resources among different sectors.

##### Estimation of water demand for each sector

**Fig. 4:**
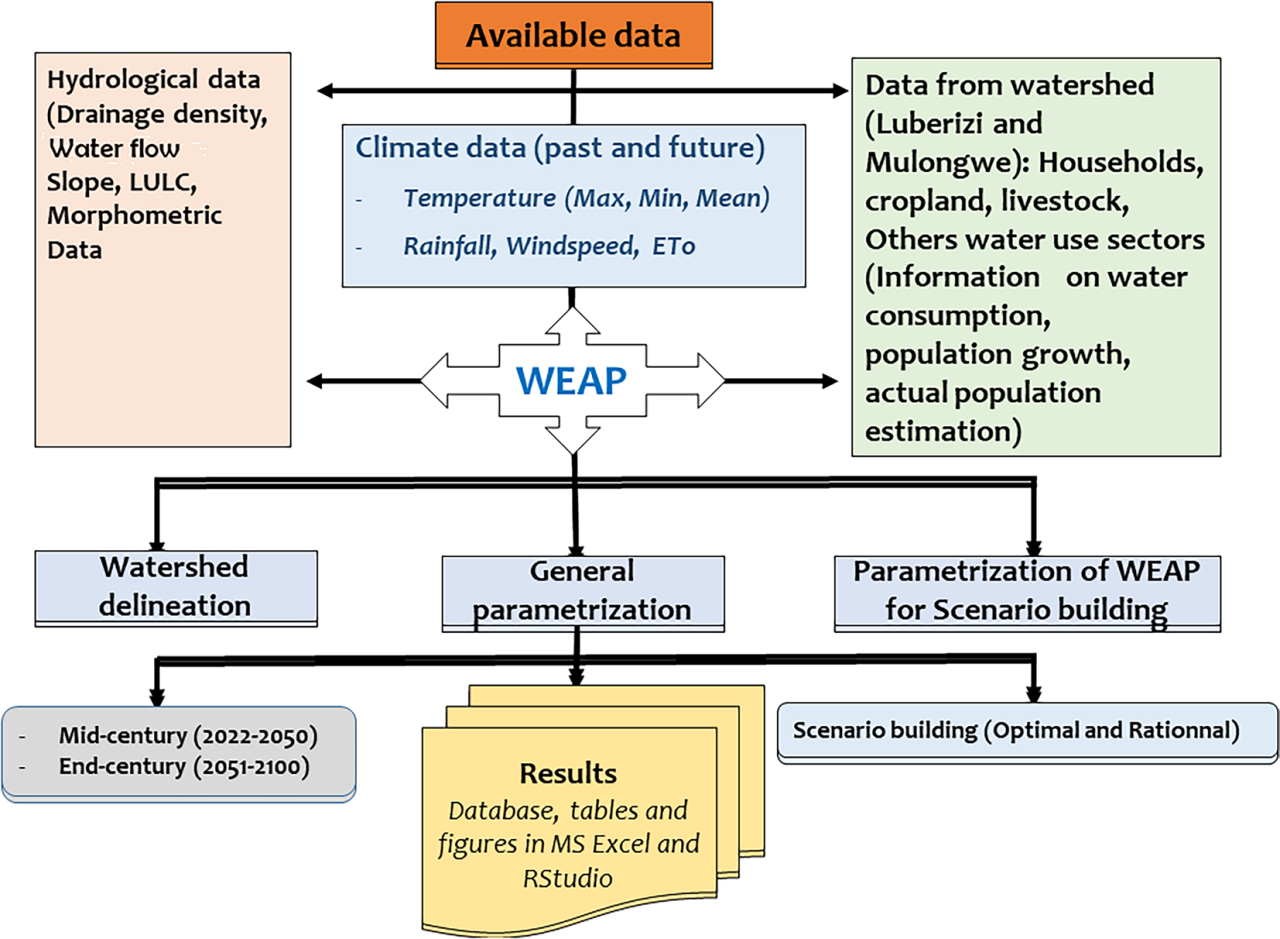
Flowchart of the methodological approach used for the application of the WEAP tool for IWRM in Ruzizi Plain, eastern D.R. Congo.

To estimate water demand in the two watersheds, Water Evaluation And Planning (WEAP) model was used. In fact, WEAP is a widely used tool for water resource planning and management^42^. Estimating water needs for different sectors in WEAP involves creating a water demand model and specifying water demands for each sector. The general steps used to estimate water needs for different sectors using WEAP are followed: (i) We first created a new model for each specific watershed; (ii) we continued to define spatial and temporal components. Here, we used the period of 2022 to 2100. The spatial components are the watersheds delineated following the process used by source in ArcGIS 10.8.1 Esri-TM and a DEM of 30 m resolution^51^. (iii) We defined demand per site for each sector within the model in considering urban areas, agricultural lands, livestock, and other uses following the data obtained from RGC (“Référentiel Géographique Commun”), the LULC, and the report for savanna used for grazing. The information for LULC is crucial for estimating water needs in the agricultural sectors. (iv) We then specified the water demand for different sectors and specified environmental flow requirements in estimating irrigation requirements based on crop types, planting calendars, and irrigation efficiency. The timing was defined next. For this study, a period from 2022 to 2100 was considered, subdivided into two subsets: from 2022 to 2050 (mid-century) and from 2051 to 2100 (end of the century). For future climate, data from Coordinated Regional Climate Downscaling Experiment “CORDEX” (CORDEX-CMIP5) and one future scenario (RCP 4.5) were selected. For each main river in the watershed, the discharge at the outlet was obtained from calculation data and fieldwork. (a) For urban built-up areas (Mulongwe), we estimated a withdrawal of 75 m^3^/ha/year (~ 200 l/day/person), a consumption-to-withdrawal ratio of about 20%, a loss of 15%, and a priority level of 2. The population growth rate used here is 4%, expressed as a growth rate of 0.04^59^. (b) For built-up areas in rural settings (Luberizi), with the population was estimated at 25,462 inhabitants, the withdrawal was estimated at nearly 12 m^3^/person/year (~ 32 l/day/person). Recognizing the pressing water access challenge in rural African and Congolese settings, these areas were given priority level 1. The consumption-to-withdrawal ratio was maintained at 7, while the growth rate was 3.5% (expressed as a growth rate of 0.035)^60^. (c) The agricultural zones used corresponded to the surface area of agricultural zones (obtained from land use data of 2022). The irrigation water withdrawal rate is 200 m^3^/ha/year^60^ (https://www.globalwaters.org/sites/default/files/drc_country_profile_final.pdf). The consumption-to-withdrawal ratio is estimated at 80%, with a priority level of 1 for Luberizi and 3 for Mulongwe. The growth rate was kept constant (1%) for both watersheds. For other uses (livestock, forests, etc.), intermediate values were used. Intermediate values were also used for other sectors (industrial, schools, churches, etc.). Figure 4 shows the flowchart of the developed methodology.

##### Evaluation of the scenarios (optimistic, rational, and reservoir creation) of water management

Three scenarios were tested: (i) the optimal scenario allowed studying the impact on demand of a policy implementing new irrigation techniques and reducing drinking water consumption, (ii) while the rational scenario comes from the baseline and maintains the same inputs regarding users and supply but with a decrease in daily allocation. Finally, (iii) the third scenario is based on the creation and consideration of wetlands as natural reservoirs for water to be used. At the end, a comparison between the scenarios was made to gain insights on available water resources in real-time and their temporal evolution based on different parameters, including demand and supply. (a) The first proposed scenario assumes significant population growth beyond the initially predicted values, reaching up to 5% for rural areas and 4% in urban areas. Since the zone is more suitable for agricultural activities (mainly rice cultivation), in rural areas, agricultural activities would increase by 3–4% instead of 1%, and a reduction to stabilization (< 0.1%). (b) The second scenario takes into account the expected impacts of current observed climate changes and disruptions, leading to a 10% annual increase in water withdrawal per person or per hectare. This scenario assumes a reduction in “Water Use Efficiency (WUE)” and a shift towards more rain-fed agriculture. This scenario also assumes that a reduction of ~ 25% would be observed in the flow of the main river due to less abundant rainfall. (c) The last scenario assumes that the presence of rich wetland areas considered here as the main source of water storage are treated as reservoirs. The policy of conserving these areas and their construction is expected to begin in 2023. Based on estimates of the wetland areas in the zone as mapped and delineated by Chuma et al.^50^, by considering only the areas within the watershed scale, a total volume of ~ 1.2 million m^3^ of water and a volume not available for allocation of 0.06 million m^3^ are obtained. Validating WEAP model outputs typically involves several steps to ensure the accuracy and reliability of the results. We have only calibrated it, analyzed the scenarios and used the peer review (to seek the feedback and validation for local and other experts in the field to ensure the structure of the model). Relevant literature on the WEAP model was also checked.

### Data sources, treatment, and analysis

Various data treatments and analyses were conducted using Microsoft Excel 2016 software, facilitating the generation of graphs and tables. Additionally, ArcGIS 10.7 Esri-TM software was employed for satellite image analysis and the creation of various maps. The use of the WEAP 2022.0 tool allowed modeling water resources and the analysis of different scenarios in the study watersheds, aiming to ensure sustainable management of these resources. The package RClimDex 1.9 from RStudio was used for climate data analysis. Correlation test, Mann–Kendall test, and Sen Slope tests were made at 5% *p*-value threshold.

## Results

### Luberizi and Mulongwe watersheds’ morphological and hydrological characteristics

The characteristics of the two selected watersheds considered for the case studies are presented in Table 1. It emerges from these two watersheds that they differ from each other. The Luberizi watershed (176 km^2^) is larger than Mulongwe (115 km^2^). These two basins have an elongated shape (Kc greater than 1), and the average slope of the main river is higher at Mulongwe than at Luberizi. This also affects the elevation difference, which is also higher at Mulongwe than at Luberizi. The number of bifurcations is higher in Luberizi (129) than in Mulongwe (85). The main river has an estimated length of 145 km (Luberizi) and 100 km (Mulongwe). These two rivers have different characteristics in terms of hydrological behaviors. Indeed, following the beginning of the river, the discharge is higher in Luberizi than in Mulongwe, partly linked to the topography and the source (highlands) of the river supplying water to the Luberizi. Additionally, the main river in the Luberizi has a larger discharge compared to the Mulongwe. In Luberizi, the highest water flow occurs in November (up to 200 m^3^/s), December (190 m^3^/s), and March (160 m^3^/s), and it is lower in the dry season (June: 30 m^3^/s, July: 25 m^3^/s). For Mulongwe, the water flow does not exceed 20 m^3^/s regardless of the season, year, or month. March, December, and April (8 m^3^/s) represent the highest flows, while June and July (less than 5 m^3^/s) represent the lowest flows. It is noteworthy that this river presents a high risk of flooding though it has a low flow. The historical data shows excessively high water flow values causing flooding in this part of the Uvira city. Luberizi is a highly drained watershed with strong agricultural potential.

**Table 1:** Morphological and hydrological characteristics of the Luberizi and Mulongwe watersheds.

Parameter	Symbol	Unit	Luberizi	Mulongwe
Perimeter	P	km	97.74	66.00
Area	A	km^2^	175.93	115.70
Gravelius coefficient	*Kc*	–	2.06	1.71
Equivalent length	Le	km	41.15	27.72
Global slope index	Ig	km/km	0.01	0.01
Mean river slope	Pmean	m/km	11.27	25.23
Denivellation	D	m	593.71	2524.00
Minimum altitude	Hmin	m	814.00	759.00
Mean altitude	Hmean	m	1691.00	2341.00
Maximum altitude	Hmax	m	3179.00	3283.00
Median altitude	H50%	m	1530.00	2442.00
Drainage density	Dd	km/km^2^	0.85	0.86
Drainage frequency	F_1_	–	0.37	0.28
Torrentiality coefficient	C_T_	–	0.29	0.32
Number of bifurcations	–	–	129.00	85.00
Main river total length	–	km	145.09	100.03
Drainage order 1	LR1	km	65.00	45.07
Drainage order 2	LR2	km	30.00	28.00
Drainage order 3	LR3	km	21.00	13.50
Drainage order 4	LR4	km	29.00	14.00

**Fig. 5:**
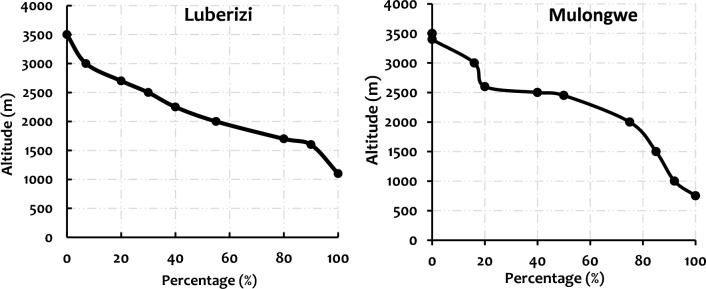
Hypsometric curves of the Luberizi and Mulongwe watersheds.

For topographic and morphological characteristics of the two watersheds, results showed that the highest points are located at ~ 3283 and ~ 3179 m a.s.l. for Mulongwe and Luberizi, respectively, while the lowest points at the outlet were ~ 759 and ~ 814 m a.s.l., respectively, for Mulongwe and Luberizi watersheds. This results in average altitudes of ~ 2341 and ~ 1691 m a.s.l., respectively, for Mulongwe and Luberizi watersheds. Hypsometric curve analysis showed that more than half of the Luberizi watershed was at an altitude exceeding ~ 1530 m, compared to 2442 m in Mulongwe. This interpretation of the curve also presented the longitudinal profile of the watershed and showed that only 10% of the Luberizi basin was at very high altitudes exceeding ~ 3000 m, while it went up to 20% in Mulongwe. Luberizi has a uniform shape, whereas Mulongwe is more complex with significant morphological variations. The hypsometric curves of the two watersheds are presented in Fig. 5.

### Climate hazard analysis in the Ruzizi plain, eastern DRC

**Fig. 6:**
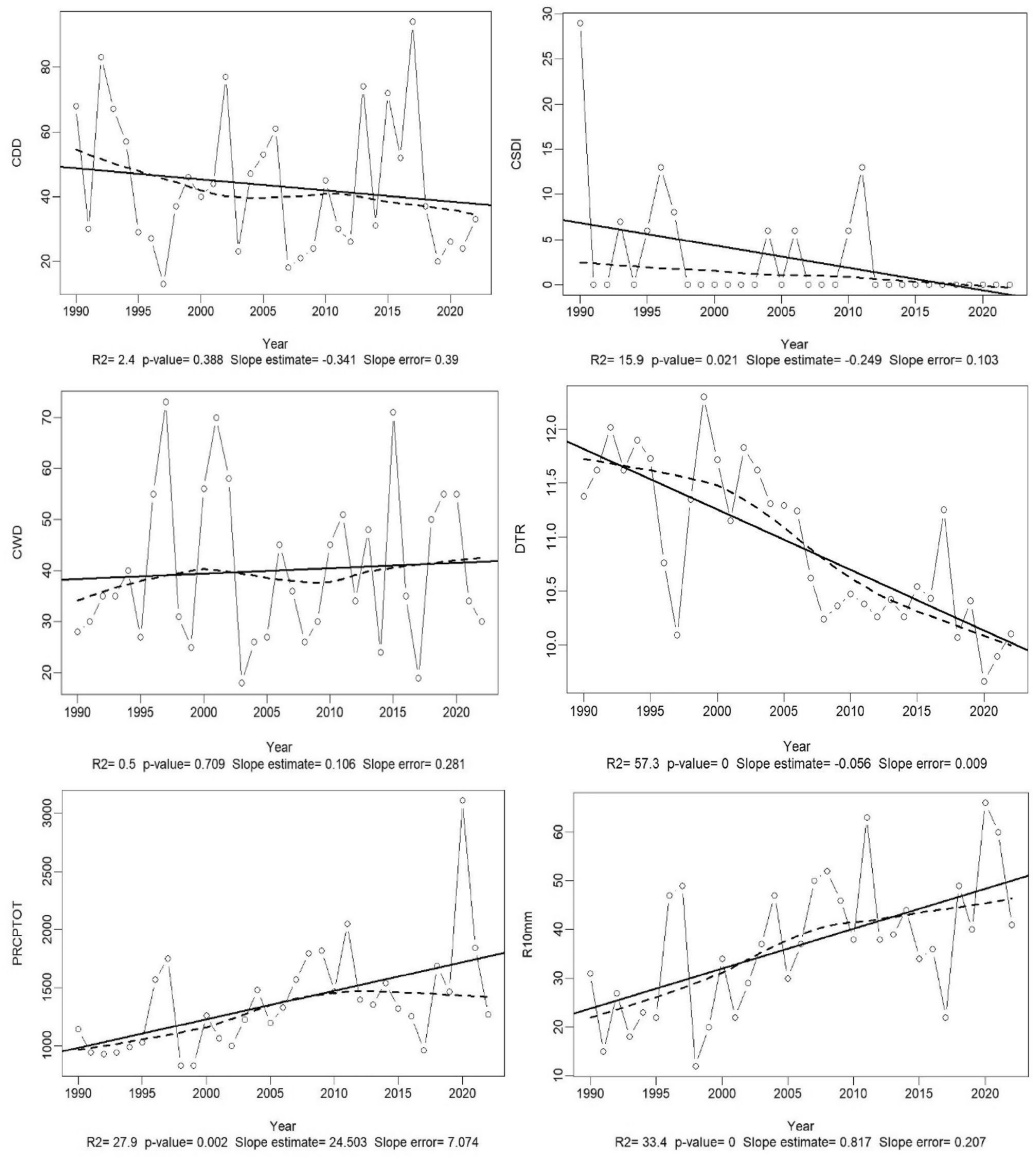

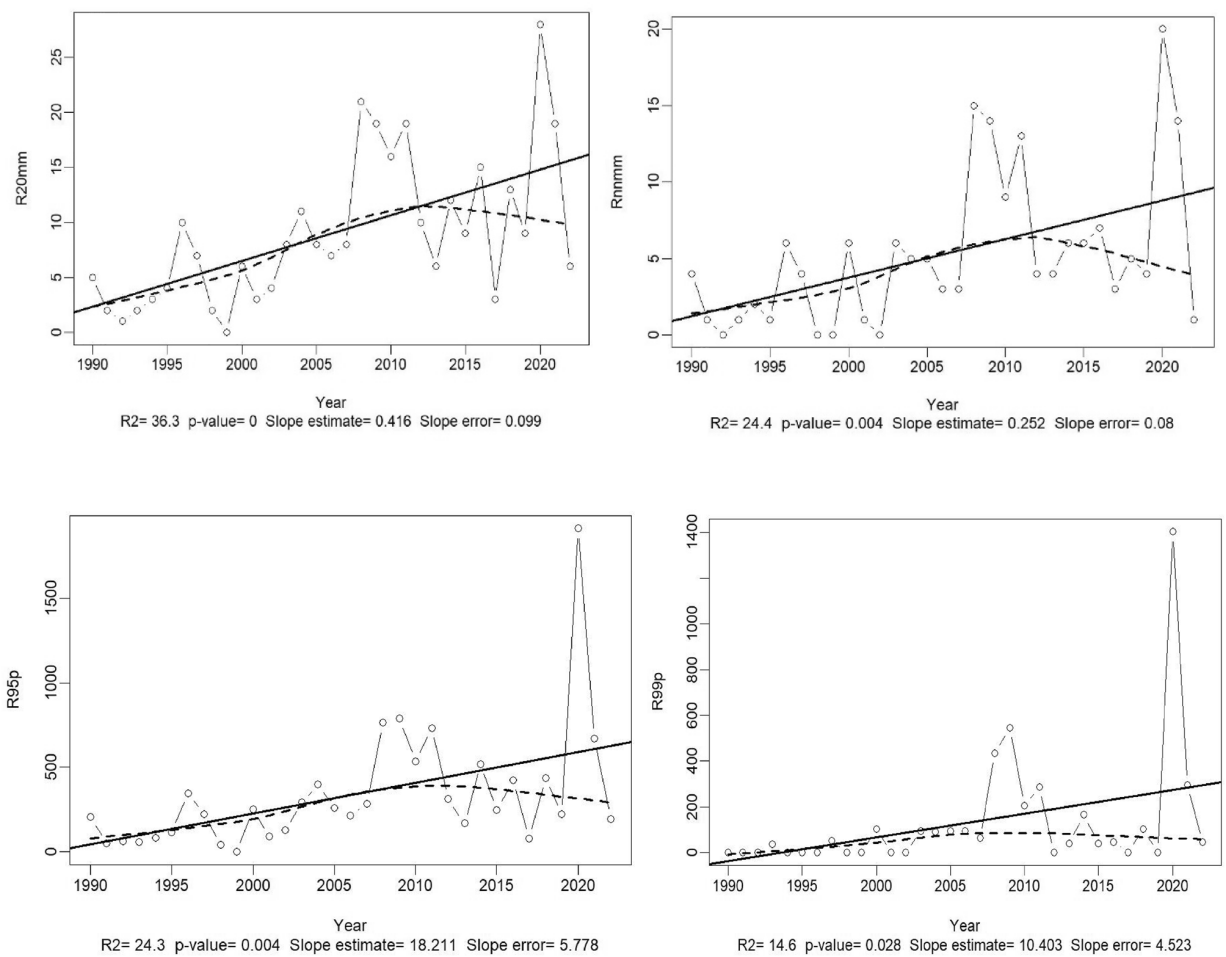
Evaluation of the trend in climate data series in the Ruzizi plain in South-Kivu, eastern D.R. Congo (Indices were calculated following the formula and processes of the RClimDex 1.9 package as developed by Zhang and Yang^53^. The explanation and description of the indices used can be found in the same article or in the Supplementary Table 1. These trends apply for both watersheds since they are located in the same climatic zones.

The results of the analysis of meteorological data from the 1990–2022 time series using the RClimDex 1.9 package are presented in Table 2 and Fig. 6. These depict the statistical analysis of the Sen Slope and the temporal trend and correlation of the selected indices. It is noteworthy that both watersheds share the same climatic realities though they mostly differ with anthropic activities. Mulongwe watershed is urban, while Luberizi is rural with significant agricultural activities. Since there is only one weather station available in the area harboring both watersheds, it was challenging to dissociate variations in climatic data between the two watersheds. More details on climate data of the Ruzizi Plain are provided by Bagula et al.^26^.

**Table 2:** Synthetic table for the climatic indices’ calculation of the used time series (1990–2022).

Indices	Starting year	Ending year	Slope	STD of slope	P-value
Tmax	1990	2022	− 0.050	0.013	0.001***
SU25	1990	2022	− 1.981	0.542	0.001***
TMin	1990	2022	− 0.063	0.020	0.004***
TMean	1990	2022	0.036	0.016	0.028*
TX10p	1990	2022	0.378	0.106	0.001***
TX90p	1990	2022	− 0.567	0.190	0.006***
TN10p	1990	2022	− 0.188	0.084	0.032*
TN90p	1990	2022	− 0.009	0.124	0.94ns
WSDI	1990	2022	− 0.663	0.349	0.067ns
CSDI	1990	2022	− 0.249	0.103	0.021*
RX1day	1990	2022	2.026	0.793	0.016*
RX5day	1990	2022	5.426	2.244	0.022*
SDII	1990	2022	0.076	0.024	0.004**
R10mm	1990	2022	0.817	0.307	0.001**
R20mm	1990	2022	0.416	0.399	0.892ns
R25mm	1990	2022	0.252	0.080	0.004***
CDD	1990	2022	− 0.341	0.390	0.388ns
CWD	1990	2022	0.106	0.281	0.709ns
R95P	1990	2022	18.211	5.778	0.004***
R99P	1990	2022	10.403	4.523	0.028*
PRCPTOT	1990	2022	24.503	7.074	0.002***

*ns* not significant: p > 0.05, significant: *p < 0.05, highly significant: **p < 0.01, and very highly significant: ***p < 0.001, *STD* standard deviation, the explanation of all the indices are presented in the Supplementary Table 1. These data are identical for both watersheds; lack of separate weather stations challenged the attempt to dissociate information.

The results show a very significant variation among certain indices across the Ruzizi plain. This is the case for the maximum temperature, the number of days when the temperature exceeds 25 °C, annual total precipitation divided by the number of wet days (defined as rainfall ≥ 1.0 mm) in the year, annual count of days when rainfall ≥ 10 mm, 20 mm, and 25 mm. The annual total precipitation when rainfall > 95th percentile and when  rainfall > 99th percentile also increased significantly (Table 2).

The interpretation of the results and trends in Fig. 6 suggest the following observations: a decrease in the number of days with precipitation less than 1 mm and the number of days with 6 consecutive days with TMax below the 10th percentile. The consecutive days with precipitation exceeding 1 mm have slightly increased over time, while the monthly difference between Tmax and Tmin (thermal amplitude) has significantly decreased. As for the number of days with precipitations exceeding 10, 20, and 25 mm (which constitute cases of extreme rainfall events with risks of flood) had increased over time. Thus, normal rainy days decreased to make way for excessively rainy days with extreme events, exposing the population to climatic hazards with all possible consequences. A very slight increase in very to extremely humid days is also observed; that is, annual precipitation where rainfall is above the 95th and 99th percentiles. The annual rainfall mean is ~ 900 mm, with some years being wetter or drier than others.

The in-depth analysis of climate data by calculating the Standardized Precipitation Index (SPI) shows that during the selected period, for every 5 dry years (negative SPI), there were series of two to three wet to very wet years. However, recent years are characterized by a reduction in this trend, with two or three dry years directly followed by one or two wet years. The trend is that after the driest year, a moderately wet year is followed by a very wet year. This trend varies very slightly from one watershed to another, with a higher drought severity recorded from the urban basin (Mulongwe) than the rural one (Luberizi). Monthly SPI analysis also shows that years tend to be subdivided into five dry months and seven wet months. The months of May and September are normal and can be either wet or dry. The same trend is observed in September and February, which are very slightly wet, so they are close to normal to moderately wet. The annual and monthly trends of the SPI indices are presented in the Supplementary Fig. 2.

### Water requirements of each sector in Luberizi and Mulongwe watersheds

#### Water demands by sector

**Fig. 7:**
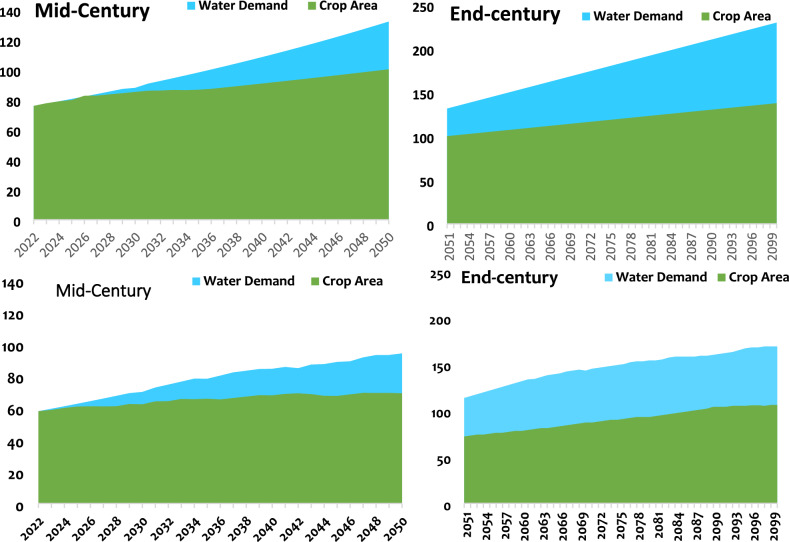
Global water demand and agricultural water demand in the Luberizi (up) and Mulongwe (down) watersheds in the mid and end of the century.

The results obtained after simulation in the WEAP software indicate a difference in total water demand between the two watersheds. These needs are higher in the Luberizi watershed than in the Mulongwe. A significant portion of water demand is required for the agricultural sector than for all other sectors. Water demand for the agricultural sector is higher in the rural watershed than in the urban one. This water demand for the agricultural sector seems to stabilize over time for the urban watershed compared to the rural one. The current water demand is estimated at ~ 76 and ~ 58 million cubic meters per year for Mulongwe and Luberizi watersheds, respectively. This demand will increase by ~ 140 to ~ 96 million cubic meters in the mid-century and ~ 246 to ~ 162 million cubic meters by the end of the century for the respective watersheds (Fig. 7).

**Fig. 8:**
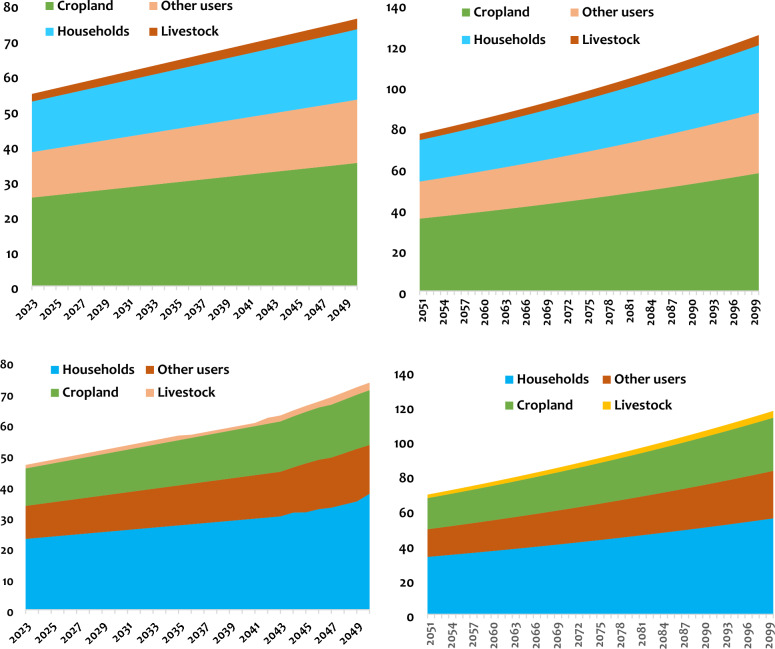
Water demands per sector in both the Luberizi (up) and Mulongwe (down) watersheds in South-Kivu province, eastern D.R. Congo.

For the agricultural sector, the demand will increase from 58 to 69 million cubic meters of water in the mid-century (an increase of up to 18% from the current demand) and up to more than 82% by the end of the century for the Mulongwe watershed. Meanwhile, for Luberizi, this water demand will increase from 76 to 100 million cubic meters, representing an increase of 31%, and up to 78% (138 million cubic meters) by the end of the century. By the end of the century, the total water demand will double in the Ruzizi Plain, with significant demands from the agricultural sector that will potentially double or even triple by the end of the century. Therefore, alternatives must be proposed to mitigate the adverse effects of these changes. Comparing the three other sectors, Fig. 8 shows that future water demand varies from one sector to another, depending on whether it is in an urban or rural watershed. For the rural watershed (Luberizi), the agricultural sector has a very high water demand, followed by other sectors, including households, with livestock presenting the lowest water demand. Conversely, for the urban-type watershed, household needs come first, followed by the agricultural sector and other sectors, with livestock once again coming last. For Luberizi, the current demand is estimated at 25 million cubic meters for the agricultural sector, 13 million cubic meters for other uses, 14 million cubic meters for households, and only 2 million cubic meters for livestock. These demands will increase by 35, 18, 20, and 3 million cubic meters by the mid-century and by 58, 30, 33, and 5 million cubic meters by the end of the century, respectively, for agriculture, household, other uses, and livestock. On the other hand, for Mulongwe, these demands will increase from 23, 11, 12, and 1 million cubic meters, respectively, for households, other uses, agriculture, and livestock in 2022 to 38, 16, 18, and 2 million cubic meters by the mid-century, and 55, 27, 31, and 4 million cubic meters by the end of the century, respectively.

#### Monthly water demand

**Fig. 9:**
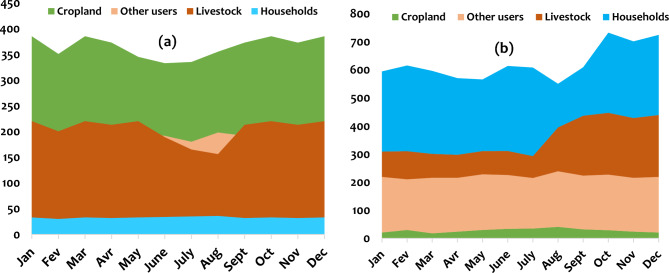
Water demand by different sectors in Luberizi (**a**) and Mulongwe (**b**) watersheds in the Uvira territory, eastern D.R. Congo.

The water demand has also been averaged per month of the year across all data sets. The results are presented in Fig. 9. By analyzing the demand by sector on a monthly basis, it appears that the demand varies from month to month. Water demands are higher in March, April, September, and October than in other months for the typical rural watershed. However, for households, these demands increase slightly in the dry season. For other sectors, the demand remains higher only in the dry season. On the other hand, for the urban watershed, the demand is more pronounced towards the end of the year, from September to December, for households and the livestock sector.

### Scenario analysis

**Fig. 10:**
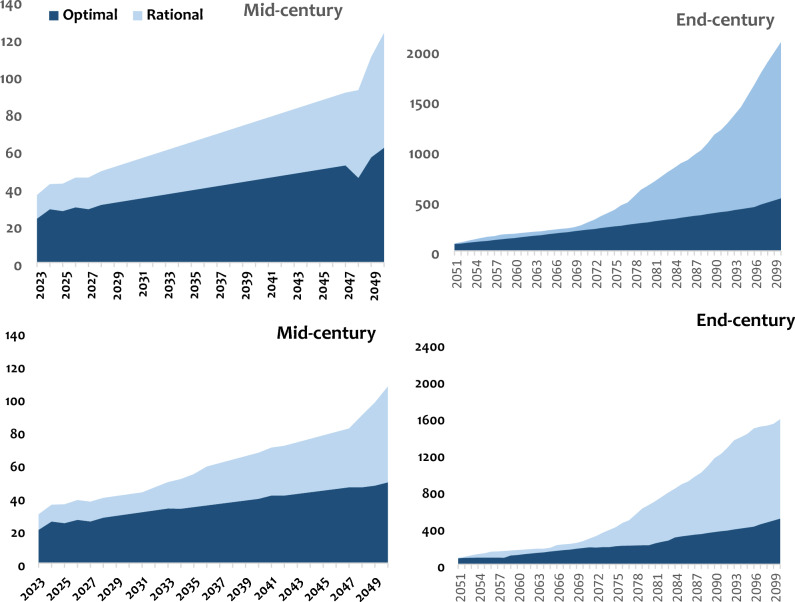
Scenarios of water management in the Luberizi (up) and Mulongwe (down) watersheds in the Ruzizi plain. The optimal scenario and rational scenarios were formulated following the description made in the methodology.

**Fig. 11:**
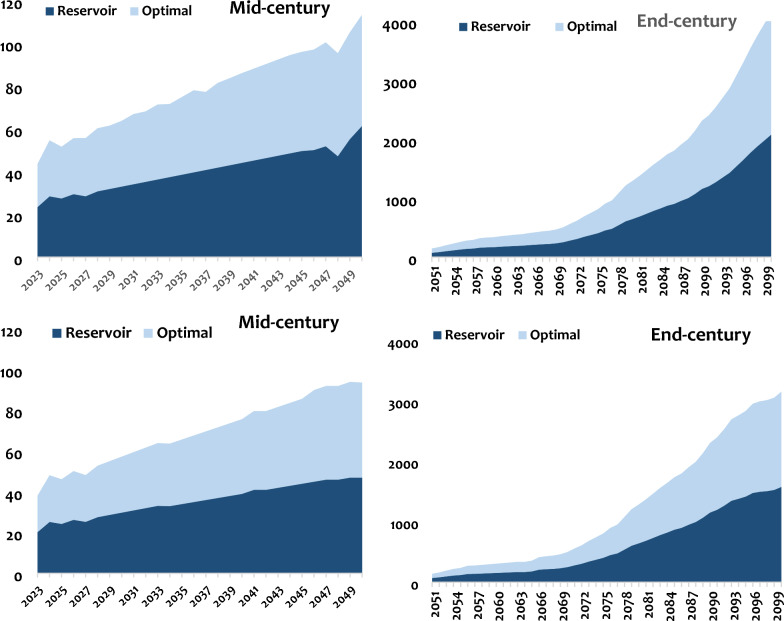
Quantification of water demands comparing the optimal scenario and the one considering wetlands as reservoirs for IWRM in Mulongwe (up) and Luberizi (down) watersheds in the Ruzizi Plain, eastern D.R. Congo.

Three scenarios were tested: one that is optimal, one rational and one that considers wetlands as water storage reservoirs for later use. The results of these scenarios are presented in Figs. 10 and 11.

From the two initial scenarios tested, the optimal water use scenario significantly reduces water demand. However, a notable demand persists until the mid-century, after which it seems to stabilize by the end of the century for the rural watershed. Conversely, for the urban watershed, water demand trend continues to increase over time until the end of the century. Between these two scenarios, the optimal scenario appears effective in stabilizing water demand, while the rational scenario leads to a substantial increase in water demand, particularly by the end of the century. Therefore, the optimal scenario is recommended for the management of both watersheds. It is essential to note that this scenario assumed significant population growth beyond the initially predicted values, reaching up to 5% for rural areas and 4% in urban areas. Given the region’s suitability for agricultural activities, especially the rice cultivation, the scenario proposes an increase of 3–4% in agricultural activities in rural areas, deviating from the initial estimate of 1%, and a reduction leading to stabilization (< 0.1%). This scenario integrated also the demand of a policy implementing new irrigation techniques and reducing drinking water consumption.

The optimal scenario was compared to the third scenario, considering wetlands as reservoirs for water storage for various uses. The results indicate that implementing reservoirs for water storage significantly reduces water demand, both in the mid-century and by the end of the century. Although the water demand will increase, the corresponding demands could reduce. This demand also appears to stabilize by the end of the century for the urban watershed, while it continues to increase for the rural watershed towards the end of the century. Therefore, considering the surface area of potential wetland areas observed in each watershed, it is evident that volumes of up to ~ 2.1 million cubic meters of water can be stored in these areas initially, increasing to a range of ~ 23 to 40 million cubic meters. Consequently, the water stored in these areas could address the water requirements of various sectors. These wetlands not only function as reservoirs but also contribute to the expansion of agricultural sectors, particularly as the majority of these areas are converted into rice fields.

## Discussion

### Watershed characteristics in Ruzizi plain

The results obtained have shown significant differences between the two watersheds selected as case studies in the Ruzizi Plain. These differences lie in their location, with one considered urban and the other rural. Morphological and hydrological characteristics have proven to be different between the two watersheds as well. The Luberizi watershed is more extensive in terms of area and perimeter compared to Mulongwe, which is located at the entrance of the Uvira city. These morphological characteristics, also known as the physical or topographic features, play a crucial role in shaping the hydrological behavior of the area^61^. They influence how water flows through the landscape and impacts the overall water resource management^62^. The size and shape of a watershed influence the amount of water captured and how quickly it responds to precipitation. Larger watersheds tend to accumulate more water, affecting downstream flow while the slope determines how water flows within the watershed. Steeper slopes can lead to faster runoff, while flatter slopes may promote water infiltration.

These results also indicate a significant change in land use and land cover in the area, characterized by a reduction or degradation of wooded areas in favor of grassy savannas and agricultural fields. This is consistent with the known spatial dynamics of the region^49,63,64^. These changes affect the water management in the Ruzizi Plain; since the type of land cover (urban, agricultural, and forest) affects the rate of water infiltration, runoff, and evapotranspiration. Urban areas, for example, often have increased runoff due to impervious surfaces^65^.

The elevation of a watershed impacts temperature and precipitation patterns and relief, or the variation in elevation, influences in general the potential energy available for streamflow^60,66^. Understanding these morphological characteristics is essential for effective watershed management in Ruzizi Plain, including flood control, water quality preservation, and sustainable land use planning. Additionally, it aids in the development of hydrological models for predicting water availability and management. Other morphological characteristics are important for water resource management. The number of bifurcations (branching of rivers) for example affects the complexity of the river network and can influence drainage patterns. The Luberizi watershed has high drainage density than Mulongwe. That indicates a well-connected river network, that affect how water is efficiently drained from the watershed.

Overall, these morphological characteristics collectively contribute to the understanding of how water moves through the landscape, affecting factors such as flooding, erosion, and water quality. Additionally, they play a crucial role in the planning and management of water resources, land use, and environmental conservation within the watershed. Utilizing these characteristics helps hydrologists and environmental planners make informed decisions for sustainable watershed management.

### Climate hazards in the Ruzizi plain

The results of the trend analysis of climate data reveal significant changes in the Ruzizi Plain. There is a decrease in normal precipitation over the past decades, giving way to days characterized by extreme rainfall events, with precipitation exceeding thresholds of 20 and 25 mm per day, as well as an increase in continuous precipitation periods exceeding five days. The analysis of the Standardized Precipitation Index (SPI) predicts months and years drier than others, with a frequency of 5 dry years followed by 2 or 3 slightly wet years. Additionally, it indicates that 4 to 5 dry months are followed by 7 wet months.

The Ruzizi Plain is facing climate hazards characterized by changing climatic patterns and increasing environmental stressors. The analysis of climate data revealed several key findings related to climate hazards in the region^22,26^. The average temperature in the Ruzizi Plain has shown variations, with potential warming trends. This has implications for various sectors, including agriculture, water resources, and ecosystems. The analysis of precipitation data indicates shifts in rainfall patterns. Some years exhibit drier conditions, with a reduction in rainfall, while others experience an increase in extreme rainfall events. This variability poses challenges for water resources and agricultural practices. As a consequence, the region is susceptible to extreme weather events, such as heavy rainfall and potential flooding. This poses risks to communities, infrastructure, and agricultural activities^67,68^. This leads to escalating water challenges, influenced by a combination of factors, including climate change, population growth, and evolving land-use patterns. This poses a threat to both water quality and quantity, increasing pressure on wetland ecosystems^22,26^.

All these elements prove that it is worth proposing a more integrated water resources policy since there are multiple challenges in water management in the Ruzizi plain for stakeholders at all levels. In general, farming practices, settlement development, and other human activities contribute to wetland degradation. Deforestation and land-use changes further affect the region’s vulnerability to climate hazards. Therefore, it is useful to propose alternatives for sustainable management. This study explored the potential of wetlands as water storage reservoirs. This scenario presents promising results in comparison to other scenarios, emphasizing the importance of considering natural features for water management. It also attempted two water management scenarios, one optimal and one rational.

Several factors explain these climate changes in the area. On one hand, significant changes in LULC observed in the entire DRC and specifically in the eastern region leading to high environmental changes. Its proximity to Lake Tanganyika and global phenomena like El Niño also explain these changes. Indeed, global changes exist, and their effects are now being felt locally^30,69,70^. It is important to remember that climate change is a complex phenomenon influenced by a combination of natural and human-induced factors though in eastern DRC, several factors have not been mentioned as contributing to climate change. Some of the factors include (i) large-scale deforestation for agriculture, tree logging, and other human activities contribute to changes in local and regional climates, since trees play a crucial role in absorbing carbon dioxide, and their removal reduces such capacity. (ii) As above-mentioned, alterations in land use, such as converting forests into agricultural land or urban areas, impact local climates through adverse effects on temperature, precipitation patterns, and overall climate stability^23,71^. (iii) Greenhouse gas (GHG) emissions from industrial activities, including energy production and processing plants, agricultural practices, such as livestock farming and rice cultivation, also produce methane, a potent GHG. (iv) Urbanization and population growth have led to increased energy consumption, waste production, and altered land use, all of which contribute to changes in local climates. Since in both rural and urban areas households are still using firewood or fuel for energy, these products are consistently in demand, thus making both substantial and marginal contributions to climate change. While human activities are significant contributors, natural factors like volcanic eruptions, solar radiation variability, and natural climate variability also play a role in climate change. The Eastern DRC is not isolated from global climate change trends. Changes in climate patterns on a broader scale, such as rising global temperatures and shifts in ocean currents, can impact regional climates.

This trend is similar to that demonstrated by Bagula et al.^26^, Muhindo^71^, and Ntole et al.^67^ in other parts (Luvungi, Sange, Kamanyola, etc.) of the Ruzizi Plain. In general, climatic trends in the Ruzizi Plain are studied and of interest to local scientists due to the agricultural and environmental significance of the plain. In fact, the area is rich in biodiversity (e.g., the Ruzizi Delta with its hippos, birds, etc.), and it is a major producer of rice, cassava, sweet potatoes, etc.^29,47,72^.

### Water management in the Ruzizi plain

The results obtained showed a high demand for water in the Ruzizi plain, with this demand varying from one watershed to another. For the selected case studies, the urban watershed presented less water demand than the rural one, but also varied among different sectors. Agricultural areas required more water than all other sectors, with this demand significantly increasing over time, doubling or even tripling by the end of the century. Given this observation, it is useful to propose alternatives to meet such a demand. Several alternatives were suggested, with a focus on a more integrated water management approach. Firstly, promoting efficient water use techniques and water conservation practices for different sectors, including agricultural zones and households, is essential^10^. This includes implementing drip irrigation techniques or other precise irrigation methods and encouraging the use of water conservation techniques in existing industrial processes in the area. For rural farmers, rainwater harvesting and conservation techniques such as Zaï pits, half-moon, ridges, etc., can be implemented^26,73^. In areas without water reservoirs, considering the construction of dams or other conservation and storage structures during significant rainy events (which are increasing in the region, as shown in the results) is advisable. Secondly, two other approaches can be mentioned: (i) raising awareness about the importance of water conservation among the local population and implementing water conservation policies and regulations for both residential and industrial users, and (ii) considering the valorization of water from the Ruzizi River or Lake Tanganyika (through desalination, for example) or investing in such research and technology, although from a socio-economic perspective, such an approach might be challenging in the area^74–76^.

**Fig. 12:**
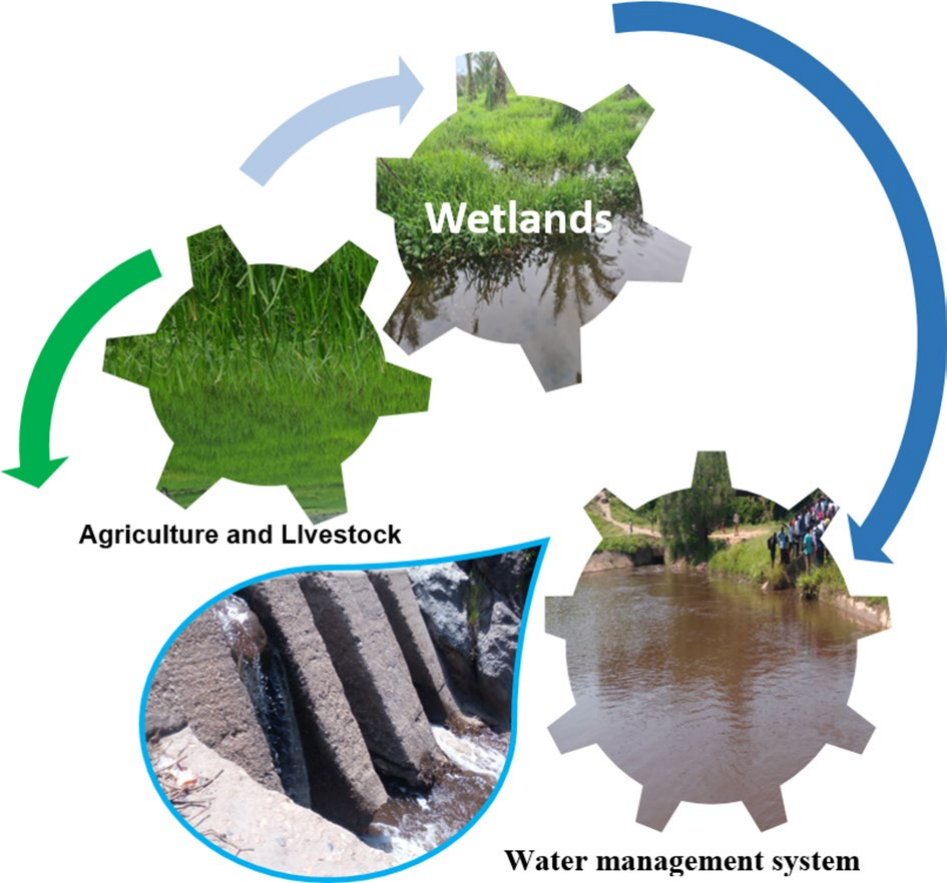
Water management in the Ruzizi plain, eastern D.R. Congo.

For our case, we focused on the wetlands of the region, which serve as true reservoirs that can help bridge the gap in water demand in the region under changing climate (Fig. 12). This, however, involves several other options. Firstly, preserving and protecting the natural ecosystems of the area, including the still-intact wetlands, which play a crucial role in maintaining water balance, and estimating the threshold of water exploitation in this area that could negatively impact local ecosystems. It will also be necessary to involve the community in such decisions while educating them about sustainable water management practices. It is also known that wetlands play a crucial role in Integrated Water Resources Management (IWRM), especially in the context of building climate-resilient futures^77^. Indeed, wetlands were considered in this study as natural reservoirs storing water during periods of excess rainfall and gradually releasing it during drier periods, acting as natural buffers against floods, contributing to groundwater recharge, and serving as biodiversity hotspots. These areas can also play other roles, such as acting as natural water filters, trapping sediments and pollutants, enhancing water quality downstream, benefiting both ecosystems and human communities. Wetlands provide recreational spaces and have cultural significance for many communities. Preserving wetlands supports the well-being of communities and maintains cultural practices tied to these ecosystems. In general, even if these zones were not extensively utilized in the area, they are nevertheless recognized that during periods of drought, wetlands can release stored water, providing a lifeline for ecosystems and downstream users. This resilience is crucial in the face of changing climate patterns, while wetlands support various livelihoods, including fishing, agriculture, and tourism, ensuring the long-term sustainability of these economic activities.

This paper adopted a forward-looking Water Evaluation And Planning (WEAP) approach, leveraging advanced modeling techniques to simulate and assess water scenarios in the Ruzizi plain. This tool allows for a comprehensive analysis of the interactions between climate, land use, and water resources, offering valuable insights for informed decision-making (Fig. 12). Through the integration of spatial data, hydrological modeling, and scenario analysis, the study aims to unravel the complex web of factors influencing water availability, distribution, and quality in the region. However, it comes from the results that the approach for a climate-resilient future in vulnerable region requires a holistic and collaborative effort, involving government agencies, local communities, businesses, and international partners to address the complex and interconnected challenges posed by climate change. Using WEAP, planners from Ruzizi Plain, eastern DRC can evaluate the resilience of water systems to climate-related uncertainties and shocks. It helps identifying vulnerabilities and design adaptive strategies to ensure water security under different climate scenarios. Lastly, WEAP plays a pivotal role in enhancing the resilience of water resources to climate change. Its capabilities in integrated modeling, scenario analysis, climate change adaptation, and stakeholder engagement make it a valuable tool for sustainable water resource management in the face of a changing climate.

By integrating climate change adaptation strategies into water management practices, such as enhancing water storage and distribution infrastructure, promoting efficient water use, and implementing nature-based solutions, we can build resilience to climate-related risks and ensure sustainable water management for present and future generations. However, the window of opportunity for action is narrowing rapidly, underscoring the critical importance of swift and decisive measures to address the urgent challenges of climate change and safeguard water resources for the well-being of both people and the planet.

The adoption of climate-smart agriculture (CSA) and the use of wetlands as reservoirs are essential adaptive measures to address the challenges of climate change. In fact, CSA involves implementing practices that enhance productivity, increase resilience to climate variability, and reduce greenhouse gas (GHG) emissions. By promoting techniques such as conservation agriculture, agroforestry, and improved water management, CSA can help farmers adapt to changing climatic conditions while mitigating their environmental impacts^52,78,79^. Additionally, utilizing wetlands as reservoirs provides a nature-based solution for climate adaptation by harnessing the water storage and regulation capacity of these ecosystems. Wetlands act as natural buffers against floods and droughts, recharge groundwater, and support biodiversity, making them valuable assets for enhancing resilience to climate change impacts. Integrating CSA with wetland management strategies offers a holistic approach to climate adaptation, fostering sustainable agricultural practices and ecosystem resilience in the face of an uncertain climate future^80,81^.

## Conclusion

The knowledge of available water resources and their variations over time is a fundamental prerequisite for rigorous and economically efficient water management planning and design. This study aimed to contribute to such management in the Ruzizi Plain, a water-scarce area. Through the characterization and evaluation of water demand in various human sectors within selected watersheds, it is evident that the Ruzizi Plain watersheds, given their characteristics, play a crucial role in the hydrological behavior of the area. The WEAP tool facilitates integrated management planning among water resources, stakeholders or users, and available resources, determining needs for the three main human sectors in the area (agriculture, households, livestock), and other users. Results showed that climate change is real in the Ruzizi Plain with the risk of pressuring wetlands to compensate currently observed water deficits for main human sectors if necessary actions are not envisaged. This study has also been able to demonstrate the effectiveness of WEAP to assess variations in future water availability, demand, and potential stressors across selected watersheds and showed the prominence of the test scenario presenting wetlands as water reservoirs as the most promising for IWRM as opposed to optimal and rational scenarios. The results indicate that water resources available to meet the demand of these sectors decrease over the years, varying from one watershed to another and from one sector to another. Given the agricultural vocation of the Ruzizi Plain, the rural watershed recorded a high water demand than the urban one. It is clear that, with the ongoing effects of climate change and rising water demand, the demand and supply dynamics will change over time until having pronounced needs from mid-century (2050) to the end of the century (2100). However, IWRM approach remains a preferable choice to prevent various disasters resulting from poor water resource management. The scenario emphasizing wetlands in the area as water reservoirs could help alleviate the water issue in the Ruzizi Plain. Although the scenario of rational water use is ideal, it appears not feasible given the unpredictability of the political and socioeconomic situations in the region. Applying the IWRM paradigm in the Ruzizi Plain can be seen as more sustainable and resilient water management system through establishment of the environment benefit, economy, and local communities. Such a holistic approach helps addressing current challenges and preparing for future uncertainties, ensuring the long-term sustainability of water resources in the region. The main limitation from our methodology is to not incorporate future climatic data into the estimation of water needs, a weakness associated with the WEAP model that does not integrate future data according to different climate scenarios. Currently, the model only relies on the initial situation and the evolution of various actors to predict the water needs.

## Supplementary Information

Supplementary Information.

## Data Availability

The datasets used and/or analysed during the current study are available from the corresponding author on reasonable request.

## Abbreviations

IWRM: Integrated water resource management
WEAP: Water evaluation and planning
DEM: Digital elevation model
LULC: Land use and land cover
SPI: Standard precipitation index
CSA: Climate-smart agriculture
