# Supplementary material for: Effectiveness of wetlands as reservoirs for integrated water resource management in the Ruzizi plain based on water evaluation and planning (WEAP) approach for a climate-resilient future in eastern D.R. Congo
Source: Sci Rep. 2024 Sep 16;14:21577. doi: 10.1038/s41598-024-72021-x (PMC11405933; doi:10.1038/s41598-024-72021-x)
Supplement: Supplementary file 1 — Supplementary Information. [file 41598_2024_72021_MOESM1_ESM.docx]

**Supplementary materials**

**
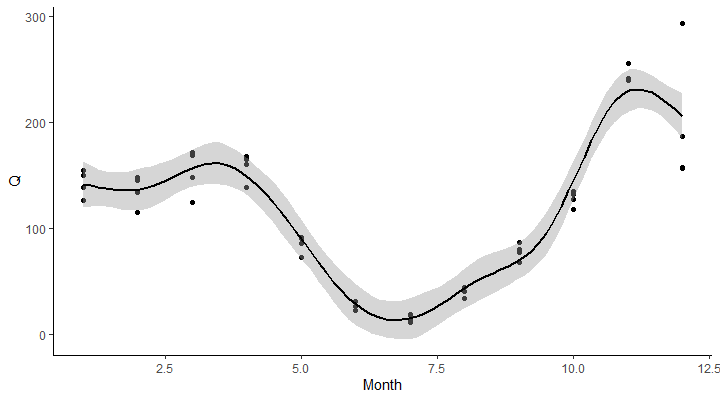
**

**
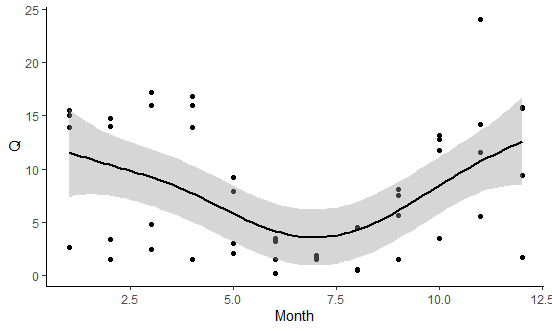
**

**Supplementary Figure 1:** Water flows (Q, m^3^/s) for the main river for Luberizi (above) and Mulongwe (below)

**Supplementary Figure 2:** Standard precipitation indices (SPI) calculated yearly (above) and monthly (below) from the data series of 1990 to 2022

**Supplementary Table 1 : Indices names, definition and units used for each indicators**

| **ID** | **Indicator name** | **Definitions** | **Units** | |  |
| --- | --- | --- | --- | --- | --- |
| FD0 | Frost days | Annual count when TN(daily minimum)<0ºC | Days | |  |
| SU25 | Summer days | Annual count when TX(daily maximum)>25ºC | Days | |  |
| ID0 | Ice days | Annual count when TX(daily maximum)<0ºC | Days | |  |
| TR20 | Tropical nights | Annual count when TN(daily minimum)>20ºC | Days | |  |
| GSL | Growing season length | Annual (1st Jan to 31st Dec in NH, 1st July to 30th June in SH) count between first span of at least 6 days with TG>5ºC and first span after July 1 (January 1 in SH) of 6 days with TG<5ºC | Days | |  |
| TXx | Max Tmax | Monthly maximum value of daily maximum temperature | ºC | |  |
| TNx | Max Tmin | Monthly maximum value of daily minimum temperature | ºC | |  |
| TXn | Min Tmax | Monthly minimum value of daily maximum temperature | ºC | |  |
| TNn | Min Tmin | Monthly minimum value of daily minimum temperature | ºC | |  |
| TN10p | Cool nights | Percentage of days when TN<10th percentile | Days | |  |
| TX10p | Cool days | Percentage of days when TX<10th percentile | Days | |  |
| TN90p | Warm nights | Percentage of days when TN>90th percentile | Days | |  |
| TX90p | Warm days | Percentage of days when TX>90th percentile | Days | |  |
| WSDI | Warm spell duration indicator | Annual count of days with at least 6 consecutive days when TX>90th percentile | Days | |  |
| CSDI | Cold spell duration indicator | Annual count of days with at least 6 consecutive days when TN<10th percentile | Days | |  |
| DTR | Diurnal temperature range | Monthly mean difference between TX and TN | ºC | |  |
| RX1day | Max 1-day precipitation amount | Monthly maximum 1-day precipitation | mm | |  |
| Rx5day | Max 5-day precipitation amount | Monthly maximum consecutive 5-day precipitation | mm | |  |
| SDII | Simple daily intensity index | Annual total precipitation divided by the number of wet days (defined as PRCP>=1.0mm) in the year | mm/day | |  |
| R10 | Number of heavy precipitation days | Annual count of days when PRCP>=10mm | Days | |  |
| R20 | Number of very heavy precipitation days | Annual count of days when PRCP>=20mm | Days | |  |
| Rnn | Number of days above nn mm | Annual count of days when PRCP>=nn mm, nn is user defined threshold | Days | |  |
| CDD | Consecutive dry days | Maximum number of consecutive days with RR<1mm | Days | |  |
| CWD | Consecutive wet days | Maximum number of consecutive days with RR>=1mm | Days | |  |
| R95p | Very wet days | Annual total PRCP when RR>95th percentile | | mm | |
| R99p | Extremely wet days | Annual total PRCP when RR>99th percentile | | mm | |
| PRCPTOT | Annual total wet-day precipitation | Annual total PRCP in wet days (RR>=1mm) | | mm | |
